# Novel T4ASS effector with quorum quenching activity

**DOI:** 10.1038/s41396-023-01497-8

**Published:** 2023-08-24

**Authors:** Vittorio Venturi, Cristina Bez

**Affiliations:** International Centre for Genetic Engineering and Biotechnology, Padriciano 99, 34149 Trieste, Italy

**Keywords:** Bacteriology, Bacterial genetics, Microbiome

Microbiota that colonize different ecosystems are most commonly diverse and populated by different bacterial taxa. These taxa rely on several mechanisms of interactions to cooperate or compete for space and limiting nutrients [1]. These mechanisms are also prevalent in plant microbiomes, typically dominated by several *Proteobacteria* members which may constitute up to 50% of the plant-associated bacterial community [2]. In the last two decades, many mechanisms of cell-cell interactions in bacteria have been discovered [3, 4]. However, a major knowledge gap exists regarding the precise mechanisms underlying the formation and maintenance of microbial communities.

Bacterial cell-cell interactions may occur via contact-independent cell-cell communication systems using diffusible molecules, such as quorum sensing signals, as well as via contact-dependent interactions using different structures like vesicles, filaments and secretion systems [4]. These latter different structures function as a channel for the exchange of biologicals including proteins, nucleic acids, signal molecules and metabolites, which affect bacterial viability or behaviour [5]. Several secretions systems can transport effector proteins into eukaryotic cells to modulate cellular functions and processes or into other bacteria in order to kill them or affect their growth. For this reason, interbacterial effector proteins most often co-occur with cognate immunity protein, which recognize and neutralize the effector protein avoiding self-intoxication [6]. Secretion systems involved in interbacterial killing (e.g. Type IV and Type VI systems) have significant ecological implications by influencing the composition, diversity and stability of microbial communities.

**Fig. 1:**
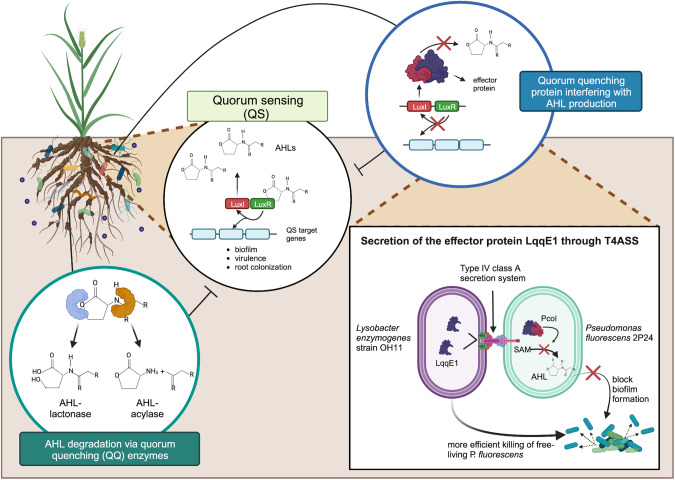
Role of the LqqE1 in plant root colonising *Lysobacter enzymogenes* strain OH11. Quorum sensing via *N*-acyl homoserine lactone (AHL) quorum sensing (QS) LuxI family synthase and LuxR sensor regulated proteins is depicted. QS interference via quorum quenching (QQ) proteins has been thus far attributed mainly to lactonase and acylase type enzymes. The T4ASS LqqE1 is translocated to target bacteria and inactivates AHL*-*QS LuxI family synthase proteins (in the Fig. 1 it is PcoI of *Pseudomonas fluorescens* 2P24) by interfering the binding with the *S*-adenosyl-L-methionine (SAM) substrate. This novel QQ activity affects QS-regulated biofilm formation allowing the target bacteria to be more susceptible to killing by other T4ASS effectors.

Liao et al. [7] explored the role of a set of effectors of a Type IV class A secretion system (T4ASS) in beneficial plant rhizosphere *Lysobacter enzymogenes* strain OH11. Their interest was whether any of the 16 putative T4ASS effectors could be injected in a rhizosphere *Pseudomonas* strain and antagonize the *N*-acyl homoserine lactone (AHL) quorum sensing (QS) cell-cell diffusible signalling system. The results of this study provide important novel insights regarding the role of effectors. AHL QS is the most common cell-cell bacterial language occurring in proteobacteria consisting of two proteins, a LuxI-family AHL signal synthase (signaling module; synthesizes the AHL), and a cognate AHL-binding LuxR-family receptor (sensing module). At “quorum” (commonly associated with a threshold of cell density) concentrations, AHLs interact directly with the cognate LuxR-family transcriptional regulators (located in the cytoplasm), binding to specific DNA motifs upstream of target genes, and resulting in the modulation of gene transcription of QS target loci [8]. Many Gram-negative pathogens use AHL QS to regulate the expression of virulence factors and biofilm formation, hence it is regarded as a potential target to mitigate or control host infections. Interference in QS is known as quorum quenching (QQ), which results in the inhibition of QS using chemical or enzymatic means counteracting behaviors regulated by QS. QQ has been the subject of extensive research, which have shown that several enzymes and chemicals may act as QS inhibitors [9]. Previous studies have reported several types of QQ enzymes including AHL-acylases or AHL-lactonases, which degrade AHLs, as summarized by Fig. 1. This degradation results in AHLs losing the capacity to act as ligands for the LuxR-family regulators, leading to the inactivation of QS (Fig. 1). Thus, QQ processes also represent a form of interbacterial interaction as they contribute to maintaining a balanced microbial community and may influence the dynamics of these populations. Liao et al. [7] identified an effector which is injected via the T4ASS into neighbouring bacterial cells and blocks AHL synthesis via binding to the LuxI-family AHL synthase. Importantly, this is the first example of anti-QS effector and of a QQ protein which is not involved in AHL degradation, but instead interfering with AHL synthesis and for this reason merits attention (summarized in Fig. 1).

The bacterial T4ASS effector, designated as LqqE1 (*Lysobacter* quorum-quenching effector 1), was shown to be translocated into *Pseudomonas fluorescens* 2P24 and inactivating the LuxI family AHL synthase PcoI [7] (Fig. 1 depicts this role of LqqE1). As *L. enzymogenes* OH11 does not possess an AHL QS system, LqqE1 hence has no protein target and is harmless in its endogenous harbouring strain (strain OH11 possesses a different QS system that utilises another type of signal). The study also reports that, mechanistically, LqqE1 impaired the ability of the AHL synthase PcoI to recognize/bind to *S*-adenosyl-L-methionine (SAM). SAM is one of the two substrates that LuxI-family synthase binds to synthesize AHLs. The LqqE1 effector was also shown to target LuxI AHL synthases of other bacterial species such as *P. aeruginosa* and *R. solanacearum*. This provides direct evidence that the mode of action of this effector is not specific to PcoI of *P. fluorescens* 2P24, but general to other LuxI enzymes. LqqE1 disables the RhlI AHL synthase of the human opportunistic pathogen *Pseudomonas aeruginosa* and the RasI of the plant pathogen *Ralstonia solanacearum*. The LqqE1 protein is therefore a non-canonical novel AHL quencher, due to the inability to directly degrade AHLs, but interfering with AHL synthesis. The study has eloquently demonstrated the ability of *L. enzymogenes* OH11 to interfere with AHL QS leading to interspecies QQ. The results have ecological implications since it confers to strain OH11 a LqqE1-dependent competitive advantage when grown in co-culture with *P. fluorescens* 2P24.

T4SS translocates toxic effector proteins into bacterial competitors inducing cell death [10]. On the other hand, the LqqE1 effector has a QQ effect without killing the competitor strain. However, this effector increases the sensitivity of the target bacterial strain. For example, target strains may use AHL QS for the formation of biofilms as a physical barrier to prevent the penetration of T4SS, and the delivery of toxic effector proteins. This series of activities ultimately result in the prevention of biofilm formation. The LqqE1 effector may enhance toxicity against free-living members rather than sessile-biofilm-bacterial-cells. This thereby confers a competitive advantage to the LqqE1 strain producer. This is particularly relevant when considering the evolutionary arms race. In this case, one possible mechanism may be targeting a regulatory systems/circuit in order to control the biofilm formation by the target strains. AHL QQ LqqE1-type effector proteins may also have major ecological implications in microbiomes and future studies will need to determine their role in biotic interactions. Figure 1 depicts these findings of the LqqE1 effector in relation to interbacterial interactions and also highlights the plant-associated ecological role and implications. Other questions that arise from this study include how widespread these types of effectors may be and the specificity towards LuxI-family synthases. This is especially true in light of the results suggesting that LqqE1 may not interfere/block the LasI AHL synthase of *P. aeruginosa*. Most mechanistic knowledge of interbacterial contact-dependent interactions via secretion systems has so far been mainly obtained using reductionist approaches such as mono/binary set-ups. It is now time to move from standard models towards more biologically pertinent ones. Scientists have recently started experimenting using synthetic communities (synComs) consisting of a set of bacteria as a simple scale representation of more complex bacterial communities. It is time to begin to capitalise on this progress and address the role of secretion systems in the context of more complex microbiomes. Understanding these mechanisms is crucial for unravelling the ecological roles of the different bacteria which constitute the plant microbiome. In addition, the benefits of understanding these mechanisms could have impacts on the development of antimicrobial strategies and the potential application of the different types of interbacterial interactions in biotechnology and biocontrol.
